# Reverse pharmacophore mapping and molecular docking studies for discovery of GTPase HRas as promising drug target for bis-pyrimidine derivatives

**DOI:** 10.1186/s13065-018-0475-5

**Published:** 2018-10-22

**Authors:** Sanjiv Kumar, Jagbir Singh, Balasubramanian Narasimhan, Syed Adnan Ali Shah, Siong Meng Lim, Kalavathy Ramasamy, Vasudevan Mani

**Affiliations:** 1 Faculty of Pharmaceutical Sciences, Maharshi Dayanand University, Rohtak, 124001 India; 2 Faculty of Pharmacy, Universiti Teknologi MARA (UiTM), 42300 Bandar Puncak Alam, Selangor Darul Ehsan Malaysia; 3 Atta-ur-Rahman Institute for Natural Products Discovery (AuRIns), Universiti Teknologi MARA, 42300 Bandar Puncak Alam, Selangor Darul Ehsan Malaysia; 4 Collaborative Drug Discovery Research (CDDR) Group, Pharmaceutical Life Sciences Community of Research, Universiti Teknologi MARA (UiTM), 40450 Shah Alam, Selangor Darul Ehsan Malaysia; 5 Department of Pharmacology and Toxicology, College of Pharmacy, Qassim University, Buraidah, 51452 Saudi Arabia

**Keywords:** PharmMapper, Bis-pyrimidine derivatives, GTPase HRas, Docking study, HEK-293

## Abstract

**Background:**

Pyrimidine is an important pharmacophore in the field of medicinal chemistry and exhibit a broad spectrum of biological potentials. A study was carried out to identify the target protein of potent bis-pyrimidine derivatives using reverse docking program. PharmMapper, a robust online tool was used for identifying the target proteins based on reverse pharmacophore mapping. The murine macrophage (RAW 264.7) and human embryonic kidney (HEK-293) cancer cell line used for selectivity and safety study.

**Methods:**

An open web server PharmMapper was used to identify the possible target of the developed compounds through reverse pharmacophore mapping. The results were analyzed and validated through docking with Schrodinger *v9.6* using 10 protein GTPase HRas selected as possible target. The docking studies with Schrödinger validated the binding behavior of bis-pyrimidine compounds within GTP binding pocket. MTT and sulforhodamine assay were used as antiproliferative activity.

**Results and discussion:**

The protein was found one of the top scored targets of the compound **18**, hence, the GTPase HRas protein was found crucial to be targeted for competing cancer. Toxicity study demonstrated the significant selectivity of most active compounds, **12**, **16** and **18** showed negligible cell toxicity at their IC_50_ concentration.

**Conclusion:**

From the results, we may conclude that GTPase HRas as a possible target of studied bis-pyrimidine derivatives where the retrieved information may be quite useful for rational drug designing.
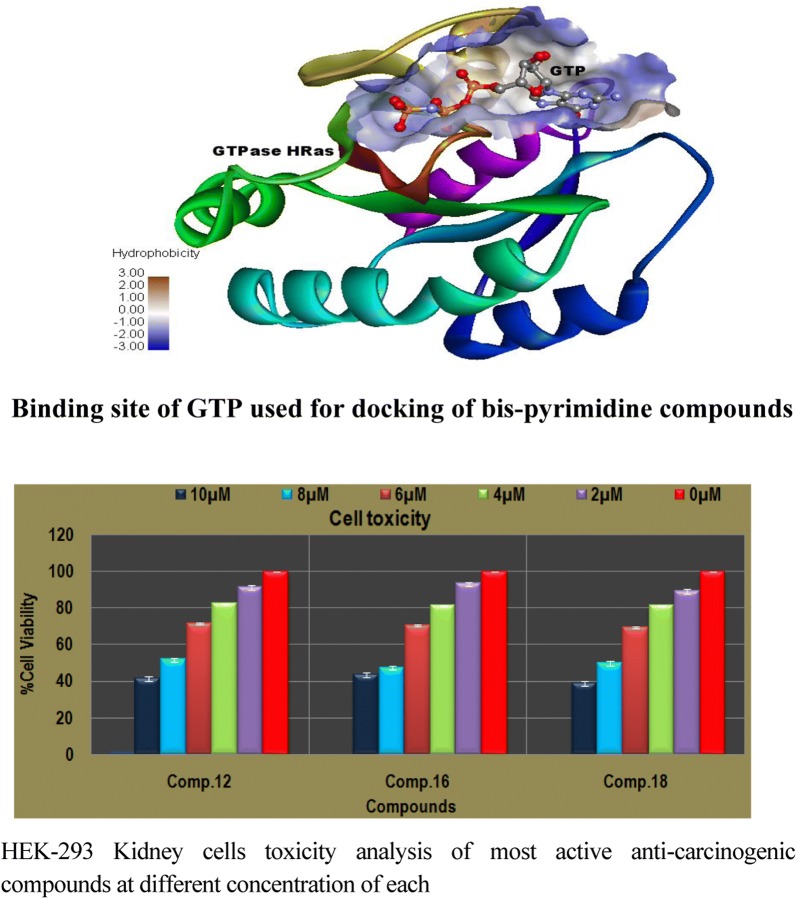

## Background

Pyrimidine is an important pharmacophore in the field of medicinal chemistry and exhibit a broad spectrum of biological potentials. Cancer, which is life threatening in nature, remains as one of the most serious global health problems. Researchers have been struggling to find effective clinical approaches for treatment of cancer over the past several decades. As such, the search for novel anticancer agents is necessary. In this regard, heterocyclic bis-pyrimidine compounds, which had exhibited potent antiproliferative activity against human colorectal carcinoma cancer cell line (HCT116) may be suitable candidates [1].

Structure-based pharmacophore modeling can effectively be used when there is insufficient information on ligands that had been experimentally proven to block or induce the activity of a particular therapeutic target. It can also be used to extract more information from the receptor side that can provide deeper insight to the medicinal chemists [2]. Molecular docking studies provide the most detailed possible view of drug–receptor interaction and have created a new rational approach to drug design [3].

Ras belongs to the family of small G proteins with intrinsic GTPases activity that governs various cellular signal transduction pathways. Ras proteins couple cell-surface receptors to intracellular signaling cascades that are involved in cell proliferation, differentiation and development. Signal propagation through Ras is mediated by a regulated GTPase cycle that leads to active and inactive conformations with distinct affinity for downstream effectors. Ras mutants with an impaired GTPase activity that are insensitive to the action of GAPs and GEFs could result in prolonged downstream signaling associated with oncogenic cell growth in diverse human cancers and leukemia. Ras genes encode multiple isoforms of which H-, N-, and K-Ras are the most abundant [4]. The Ras isoforms, H-Ras are GTPases that play important roles as regulators of signal transduction pathways that are involved in cell growth, differentiation, migration and apoptosis. All Ras proteins are anchored to the membrane via posttranslational modifications at their C-terminal hyper variable regions (HVR) that guide localization into distinct membrane compartments [5].

Based on the facts mentioned above, reverse docking was used in the present study to identify the drug target of anticancer bis-pyrimidine derivatives (identified in an earlier study) using PharmMapper web server. GTPase HRas yielded better fitness score and have also been found as an important drug target against cancer earlier. The specificity for identified target was assessed with docking using Schrodinger *v9.6.* The study concluded the possibility of GTPase HRas as drug target of bis-pyrimidine derivatives and druggability of GTP binding site.

## Results and discussion

### Data set

The data set of bis-pyrimidine derivatives (**1**–**20**), which exhibited selective antiproliferative activity against human colorectal carcinoma cancer cell line (HCT116) (IC_50_ = ranging from 0.73 to 4.16 µmol/mL) but not showed significant results against murine macrophage cell line (RAW 264.7) (IC_50_ = ranging from 3.50 to 4.16 µmol/mL) (Table 1) were selected from the literature for development of the pharmacophore model. The selected data set are shown in Table 1 [1].

**Table 1:**
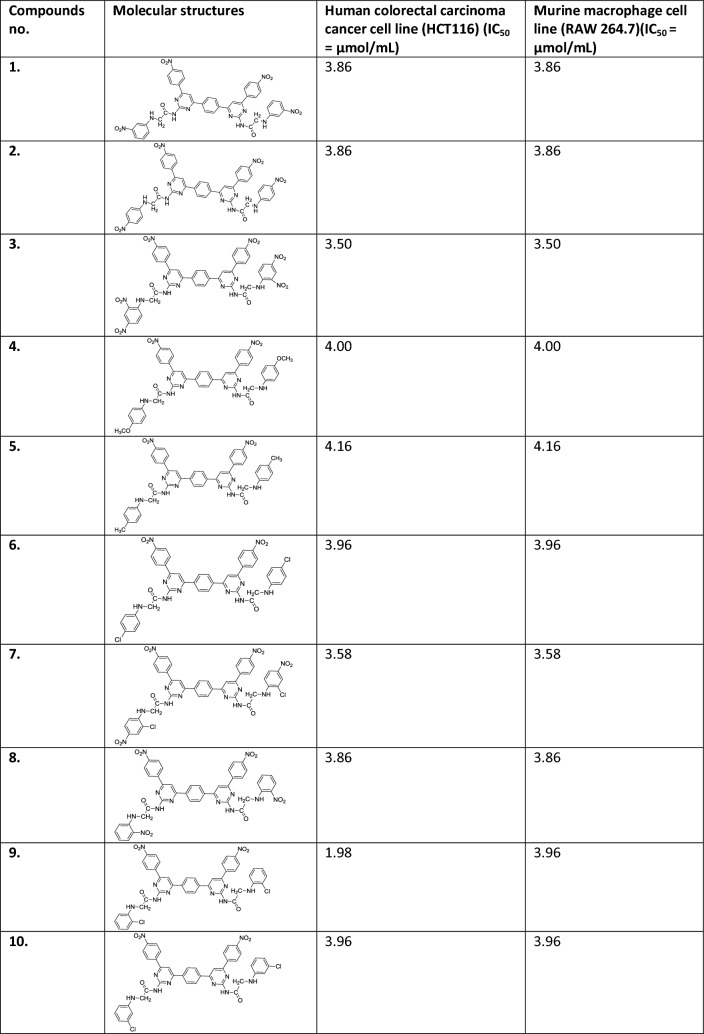

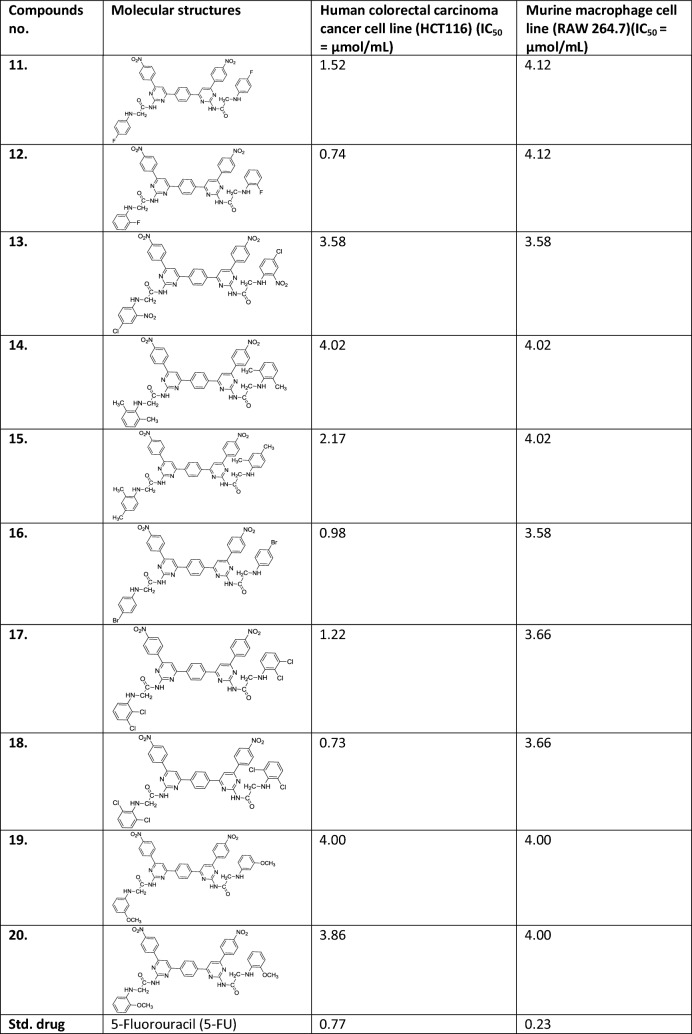
The selected data set of bis-pyrimidine derivative (**1**–**20**) against HCT116 and their antiproliferative effect against RAW 264.7

### Target identification of compounds

An open web server PharmMapper was used to identify the possible target of the developed compounds through reverse pharmacophore mapping [6]. The reverse pharmacophore mapping strategy has been used to find the protein targets of cardamom essential oils [7]. PharmMapper identifies the possible potential targets of given query (bis-pyrimidine compounds) based on the reverse pharmacophore mapping. It compares the pharmacophores of the query compounds against in built pharmacophore models database of annotated 23,236 proteins from BindingDB, TargetBank, DrugBank, PDTD with 16,159 druggable and 51,431 ligandable pharmacophore models. It provides results in form of Z score according the similarity of pharmacophore of query compounds with the identified target pharmacophore model along with importance of target protein in diseases and indications are also given [8, 9]. So the most active compound **18** was submitted to PharmMapper to identify its possible drug target. Target protein was selected based on the importance found in the development of cancer.

### Target identification

From the selected data set, compound **18** which showed the potent antiproliferative activity (IC_50_) 0.73 µmol/mL was submitted to the PharmMapper (http://59.78.96.61/pharmmapper). PharmMapper compared the pharmacophores of the most active compound **18** with the in-built database of pharmacophore models and provided the target information of 300 proteins with their fitness score and number of pharmacophoric features, indication and importance of each protein. 300 Protein retrieved were ranked according to their fitness score. Top 10 proteins with fitness score more than 5.0 were studied to identify the possible target protein of compound **18** and target selection was done based upon the importance of protein in cancer disease (Table 2).

**Table 2:** Details of top ten protein hits from PharmMapper pharmacophore mapping

S. no.	Protein name	PDB Id	Disease	No. of pharmacophore features	Fitness score
**1.**	Streptavidin	1SRE	None	9	6.17
**2.**	Non-specific lipid-transfer protein 1	1UVB	None	7	6.105
**3.**	UPF0230 protein TM_1468	1VPV	None	7	5.822
**4.**	Aspartate aminotransferase	1ASG	None	9	5.443
**5.**	GTPase HRas	5P21	Defects in HRAS are the cause of costello syndrome, tumor predisposition, congenital myopathy, Hurthle cell thyroid carcinoma, thyroid cancers Bladder cancer; oral squamous cell carcinoma (OSCC)	15	5.424
**6.**	Palmitoyl-protein thioesterase 1	1EH5	None	6	5.421
**7.**	Chorismate synthase	1QXO	None	10	5.323
**8.**	Tyrosine-protein phosphatase non-receptor type 1	1Q6N	None	7	5.263
**9.**	Phospho-*N*-ethanolamine methyltransferase	3UJ9	Malariae infection	9	5.255
**10.**	Beta-lactoglobulin	1B0O	None	6	5.245

First four protein from the Table 2 were got highest fitness score but were not found to be indicated for any disease. The fifth protein GTPase HRas with fifteen pharmacophoric features (eight acceptor, five donor and two negative) (Table 3) scored fitness score 5.424 was found to have important role in causing cancer. It has been demonstrated that defects in HRas may lead to bladder, Costello syndrome etc. GTP based HRas protein is found to involve into regulation of cell division and cell growth through signal transduction. The function of the protein is controlled by the GTP where GTP is converted into GDP. Since, HRas belongs to oncogene family it can lead normal cell to be cancerous [10]. Costello syndrome is a rarely found disease in which many parts of the body are affected and get prone to be cancerous and noncancerous tumors. Much mutation in the HRas protein has been identified which are responsible for abnormal function of HRas protein triggers the cell growth signals to grow constantly and uncontrolled cell division leads to the Costello syndrome or cancer [11, 12].

**Table 3:** Pharmacophoric features of GTPase HRas protein aligned over most potent compound **18** [green: donor, magenta: acceptor, red: negative]

PDB Id	Name	Hydrophobic	Negative	Positive	Aromatic	Acceptor	Donor
5P21	GTPase HRas	0	2	0	0	8	5

Mutations into the HRas protein have also been found to be cause of bladder cancer. Mutations make cells overactive to grow and divide at abnormal rate which have found to associate with progression of bladder cancer. Over expression of this protein has been studied in the other type of cancers, so the somatic mutation found in the HRas genes is also probably associated with other types of cancer [13, 14]. The protein was found one of the top scored targets of the compound **18**. Hence, the GTPase HRas protein was found crucial to be targeted for competing cancer. Protein was further evaluated for the binding affinity for the studied bis-pyrimidine derivatives through the docking program.

### Docking

**Fig. 1:**
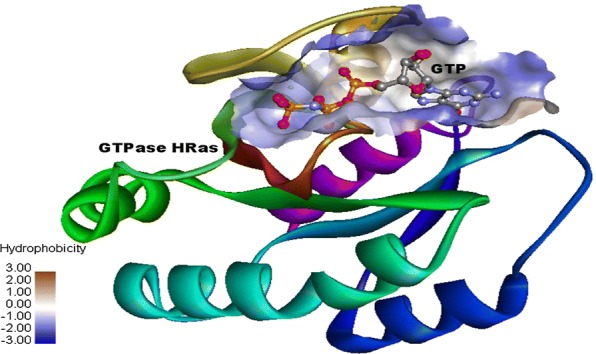
Binding site of GTP used for docking of compounds

Prior, to the docking the GTPase HRas and bis-pyrimidine derivatives were prepared and then, docked using Glide module of Schrodinger *v9.6.* While preparing crystal structure of GTPase HRas, co-crystallized water molecules within 3 Å of co-crystallized GTP were kept as retained water molecules have been found crucial for GTP binding. GTP was kept as docking control with docked score = 4.97 and binding energy = − 48.7 to score the compounds studied. The binding sites were analyzed through SiteMap and the best active site was found with site score 0.726, D-score (druggability score) 0.719 and volume 103.84. The core of binding site was found lipophilic surrounded by hydrophilic environment Active site was found over the GTP covering important amino acids of GTP binding site (Fig. 1).

**Fig. 2:**
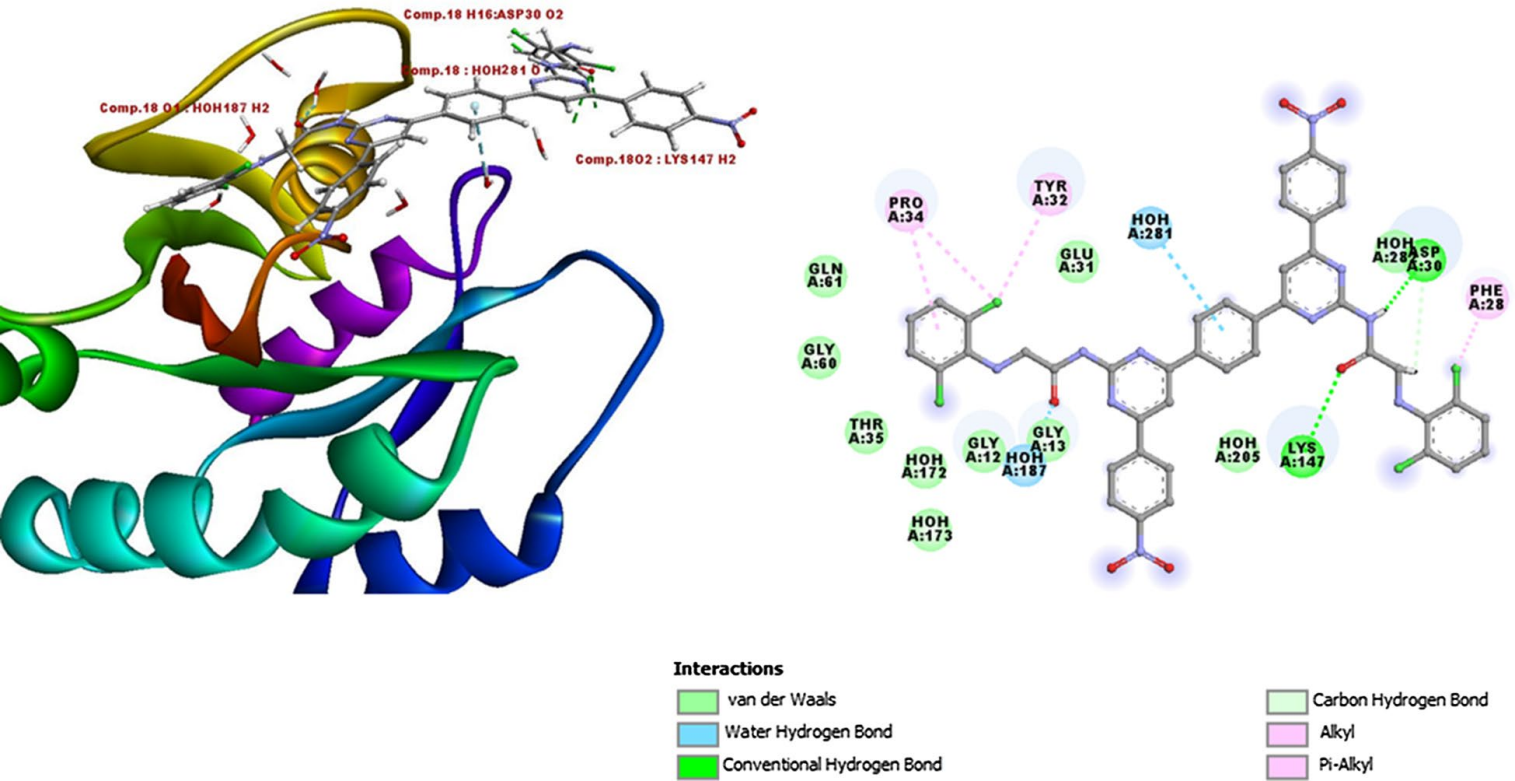
Details of binding residues and orientation of compound **18** within the GTP binding pocket and secondary structural representation of compound **18**

Hence, the binding site of GTP was created as binding site with dimensions (X = 12.5087, Y = 33.7101, Z = 19.8773) for docking of bis-pyrimidine derivatives. All the bis-pyrimidine compounds were scored via flexible docking (XP docking) where compounds were flexible and found to score better than GTP as docking control (Table 4). Minimization of docked compounds within binding site was done and most stable orientation with lowest possible energy was analyzed. Water molecules within binding site were plying crucial, formed bond with pyrimidine derivatives. If we look into the mode of binding of most active compound **18** within binding site, compound **18** scored better docked score (7.90) and binding energy (− 68.2) than GTP formed hydrogen bond with crucial Asp30 and Lys147 residues. Pro34 and Tyr32 residues were also occupied by compound **18** through Pi bonding and compound **18** was also forming van der Waals interaction with other crucial amino acids like Gln61, Gly12, Gly60 etc. which enables the close and good packing of compound into binding site compound **18** was also formed hydrogen bonds with H_2_O 187 and H_2_O 281 and van der Waals interaction which crucially binds with GTP. Binding orientation was found quite similar to the GTP binding mode within binding pocket (Fig. 2).

**Table 4:** Docking score and binding energy of bis-pyrimidine derivatives

Compound no	Docking score	Binding energy
1.	6.51	− 58.2
2.	6.10	− 54.5
3.	6.43	− 52.5
4.	7.42	− 62.6
5.	5.91	− 54.9
6.	6.86	− 62.7
7.	7.01	− 60.2
8.	6.05	− 58.4
9.	6.30	− 52.3
10.	5.70	− 56.9
11.	6.62	− 56.8
12.	7.86	− 65.5
13.	5.60	− 58.4
14.	5.91	− 58.2
15.	6.70	− 62.5
16.	8.13	− 64.8
17.	5.81	− 54.8
18.	7.90	− 68.2
19.	6.74	− 54.8
20.	7.06	− 62.6
GTP	4.97	− 48.7

**Fig. 3:**
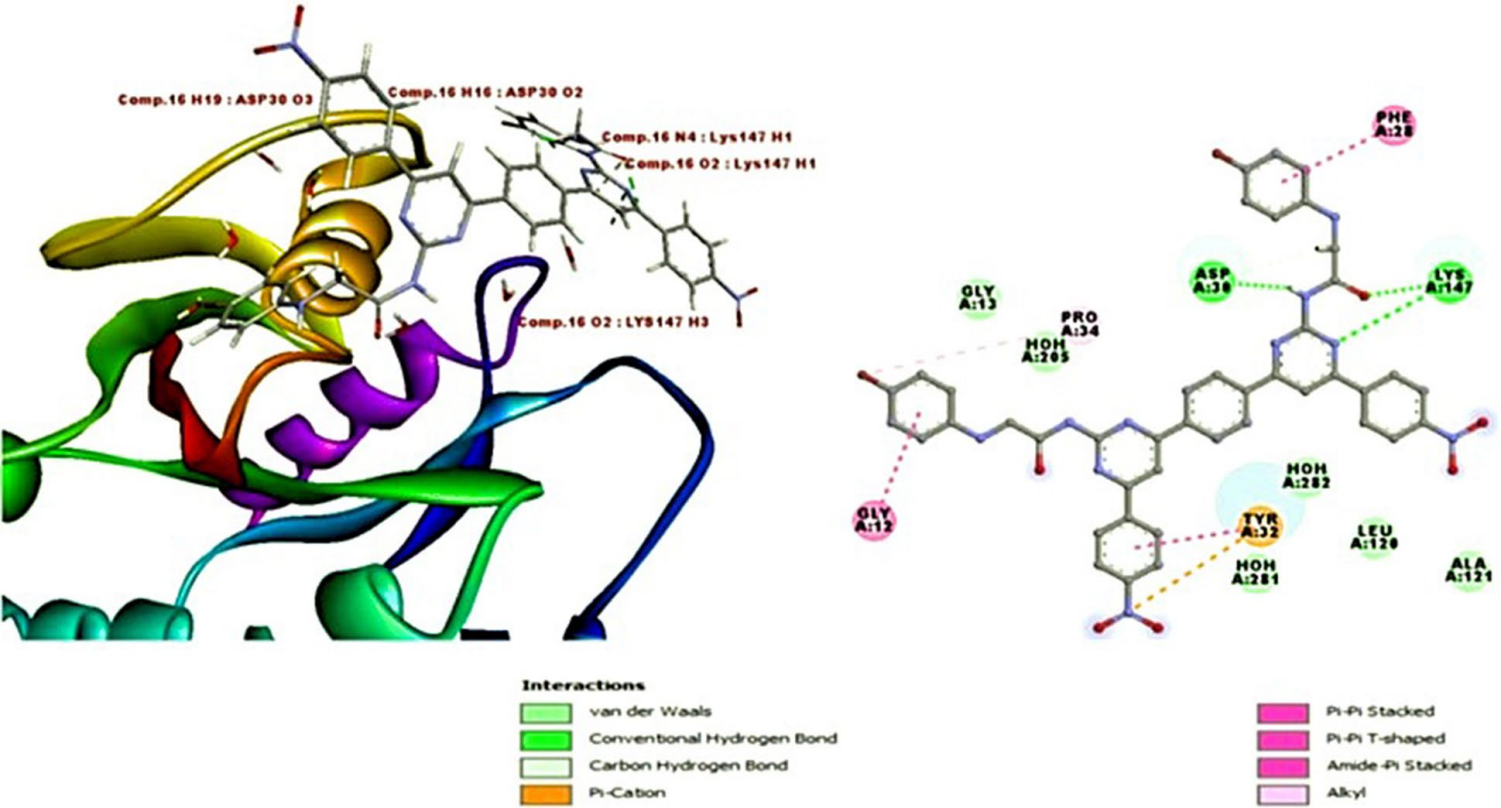
Details of binding residues and orientation of compound **16** within the GTP binding pocket and secondary structural representation of compound **16**

If we also look into binding orientation of the best scoring compound **16** with highest dock score (8.13) with better binding energy (− 64.8) was also found to bind in similar mode like GTP and most active compounds, comp. **18** and comp. **16** occupied the protein through four hydrogen bonds with crucial Asp30 and Lys147 residues. Pi–Pi interaction and Pi cation bonds were formed by the compound **16** with GTPase HRas implied the strong binding of the compound into the binding pocket (Fig. 3). The reverse pharmacophore mapping (PharmMapper) and docking results demonstrated the specificity of pyrimidine compounds for the GTPase HRas. Compounds showed better interaction and binding affinity than GTP for GTPase HRas also the lower binding energy compounds found signified thermo-dynamically stability. Hence, the GTPase HRas may be the possible target of anti-carcinogenic bis-pyrimidine derivatives studies. The experimental work will be carried to validate the affinity and mode of inhibition of compounds towards target protein.

### Antiproliferative effect against RAW 264.7

Table 1 shows the comparison of the IC_50_ values of the bis-pyrimidine derivatives (**1**–**20**) between HCT116 and RAW 264.7. The antiproliferative effect of these compounds appears to be cell type-dependent. Bis-pyrimidine derivatives (**1**–**20**) exhibited excellent selectivity of the compounds towards the human colorectal carcinoma cell line instead of the murine macrophages. The IC_50_ bis-pyrimidine derivatives (**1**–**20**) against RAW 264.7 were all beyond the highest tested concentration. The standard drug, 5-FU, exhibited antiproliferative effect against both cell lines.

### Cell toxicity analysis against HEK-293

**Fig. 4:**
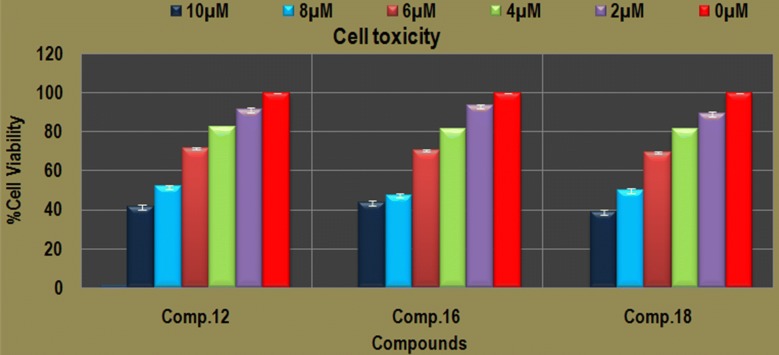
HEK-293 kidney cells toxicity analysis of most active anti-carcinogenic compounds at different concentration of each

For the selectivity index calculation of the three top dock scoring compounds, these were tested against normal human embryonic kidney cell line (HEK-293). Compounds were dissolved into 0.1% DMSO solution. The compounds were diluted in concentration (2 µM, 4 µM, 6 µM, 8 µM and 10 µM). The cells were incubated with these compounds for 24 h and more than almost 100% of HEK-293 cells were viable at IC_50_ for growth inhibition of each studied compound. Results showed the significant viability difference between the test compound treated and control cells (at zero concentration) after 24 h with (P < 0.01). The 50% of the cells were viable at the lethal dose (LD_50_) 8.53 µM, 8.41 µM and 8.21 µM of the compounds, comp. **12**, comp. **16** and comp. **18**, respectively. As we know that higher the LD_50_ value than the IC_50_ higher will be the selectivity that implied that the compounds may have better safety of the each of three compounds since the IC_50_ is much lower the LD_50_ (Fig. 4). The selectivity index of the each compound suggested the better safety of each (Table 5).

**Table 5:**
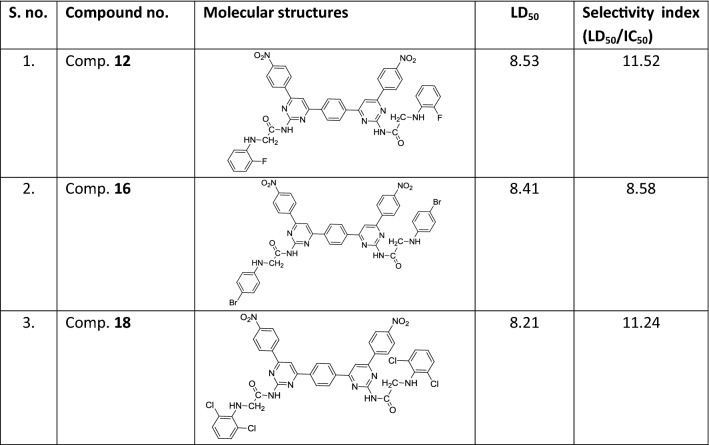
Lethal dose (LD_50_) and selectivity index calculation of most active compounds

## Experimental

### Protein preparation

Protein was prepared by protein preparation wizard where protein was preprocessed and optimized. After that OPLS 2005 force field was applied to minimize the structure. The typical structure file from the PDB is not suitable for immediate use in molecular modeling calculations. A typical PDB structure file consists only of heavy atoms and may include a co-crystallized ligand, water molecules, metal ions and cofactors. Some structures are multimeric and may need to be reduced to a single unit. Because of the limited resolution of X-ray experiments, it can be difficult to distinguish between NH and O and the placement of these groups must be checked. The preparation of a protein involves a number of steps, which are outlined below. The procedure assumes that the initial protein structure is in a PDB-format file, includes a co-crystallized ligand and does not include explicit hydrogen. The result is refined, hydrogenated structures of the ligand and the ligand–receptor complex, suitable for use with other Schrödinger products [15].

### Active site analysis and binding site creation

The top five active sites of target protein were analyzed using SiteMap under OPLS_2005 force field with default settings but the hydrophobicity of active sites was more restrictive. The active sites were scored according to their site volume and site score. The location of the primary binding site on a receptor such as a protein is often known from the structure of a co-crystallized complex. SiteMap generates information on the character of binding sites using novel search and analysis facilities and provides information for visualization of the sites. A SiteMap calculation was begin with an initial search stage that determines one or more regions on or near the protein surface, called sites that may be suitable for binding of a ligand to the receptor. The search used a grid of points, called site points, to locate the sites. In the second stage, contour maps (site maps) were generated, producing hydrophobic and hydrophilic maps. The hydrophilic maps were further divided into donor, acceptor, and metal-binding regions [16, 17]. The GTPase HRas (PDB Id: 5p21) was retrieved from PDB http://www.rcsb.org/pdb/home/home.do) for docking of bis-pyrimidine derivatives. Grid generation module of Schrodinger *v9.6* was used to generate grid of top active site which covered the important amino acids from GTP binding site.

### Ligand preparation

Ligand preparation is done using LigPrep module of Schrodinger *v9.6.* To give the best results, the structures that are docked must be good representations of the actual ligand structures as they would appear in a protein–ligand complex. This means that for Glide5.5 docking the structure must meet the following conditions. They must be three-dimensional (3D). They must have realistic bond lengths and bond angles. Glide only modifies the torsional internal coordinates of the ligand during docking, so the rest of the geometric parameters must be optimized beforehand. They must each consist of a single molecule that has no covalent bonds to the receptor, with no accompanying fragments, such as counter ions and solvent molecules. They must have all their hydrogens (filled valences). They must have an appropriate protonation state for physiological pH values (around 7). Protonation states are particularly crucial when the receptor site is a metalloprotein such as thermolysin or a MMP. If the metal center and its directly coordinated protein residue have a net charge, Glide assigns a special stability to ligands in which anions coordinate to the metal center. They must be supplied in Maestro, SD, Mol2, or PDB format. Maestro transparently converts SD, MacroModel, Mol2, PDB and other formats to Maestro format during structure import [18].

### Docking

Once the target protein is identified as GTPase HRas, was used for screening of bis-pyrimidine derivatives library was screened through GTP binding site using extra precision (XP) docking module of Schrodinger *v9.6.* XP module performs docking the compounds with better precision and accuracy. The dataset size goes smaller as the docking accuracy increases at each stage [17]. The endogenous ligand Guanosine triphosphate (GTP) was used as docking control and binding energy was also calculated (PrimeMM-GBSA module) Schrodinger *v9.6* [19].

### Sulforhodamine (SRB) assay

The murine macrophage cell line (RAW 264.7) were seeded onto the 96 flat bottom well plate at 7000 cells/well and allowed to attach overnight. The cells were then exposed to the respective compounds for 72 h and subjected to the sulforhodamine (SRB) assay [20]. Treated cells were then fixed in trichloroacetic acid and stained in SRB dye (0.4% (*w/v*) SRB mixed with 1% acetic acid). The optical density of the plate was read at 570 nm using a microplate reader.

### Cell toxicity (MTT assay)

Human embryonic kidney (HEK-293) cells were maintained in Dulbecco’s modified Eagle’s medium (10% heat-inactivated FBS). Antibiotics penicillin and streptomycin were added and were placed at 37 °C in a 5% CO_2_ incubator for colorimetric based assay using MTT (3-[4,5-dimethylthiazol-2-yl]-2,5-diphenyltetrazolium bromide) comp. **12**, comp. **16** and comp. **18** were seeded with five thousand HEK-293 cells (viability 98%) into 96-well plate for 24 h. Wells were added with MTT 5 mg/mL after 24 h incubation for 4 h [21]. Absorbance at 580 nm was recorded using Synergy/HTX MultiScan reader (BioTek) and lethal dose LD_50_ was calculated and for selectivity index (SI) was calculated.

## Conclusion

Target finding of a drug or compound is difficult but a target possibly may be identified using computational approaches at minimum cost and time. Reverse docking of any compound with known druggable targets available that may provide information of interacting features and affinity of a protein for the compound. The used online server PharmMapper which works on the principle of reverse docking generates information about the pharmacophoric features of protein binding site for a compound docked. Compounds studied have already been tested with potent anticarcinogenic activity at very low µmol/mL concentrations [2]. So the target information of most potent compound **18** from PharmMapper brought the information about the possible drug targets. Among the top ten protein hit, GTPase HRas which has been crucial role in formation of tumor, Costello syndrome and other type of cancers was found one with better fitness score. The target protein helps in transmitting signal transduction from outer side to inner side of nucleus to generate new cells faster. Among top ten scored proteins provided from PharmMapper was only protein found indicated in cancer diseases. The further docking of pyrimidine compounds within the GTP binding site of GTPase HRas protein using Schrodinger *v9.6* revealed that the compounds were interacting in a orientation similar to GTP. The compounds were revealed that the compounds were interacting in an orientation similar to GTP. The compounds were also interacting with H_2_O molecules which were found important for GTP binding and hydrolysis within the binding site. Compounds formed non covalent binding with some crucial amino acids like Gly12, Val29, Asp30, Gly60, Lys117, Ala146 etc. The binding and orientation of compounds, comp. **18** and comp. **16** based on potency and docking affinity more than GTP implied the specificity towards target protein with lower binding energy. Besides, the antiproliferative effect of bis-pyrimidine derivatives (**1**–**20**) appears to be cell type-dependent. These compounds were more selective towards cancer cells rather than macrophages. In the present study, effect of most active compounds on the cell viability of non-cancerous HEK-293 cells was also examined. The results demonstrated better selectivity index against the HEK-293 cell lines at the respective IC_50_ concentration. Study suggested that compound may be safer as anticancer after required experimental evaluation. Bis-pyrimidine derivatives (**1**–**20**) exhibited excellent selectivity of the compounds towards the human colorectal carcinoma cell line instead of the murine macrophages.

Hence, study enlightened the importance of reverse docking for the prediction of target of active compounds. The utility of tools like PharmMapper which works on reverse docking for identification of possible drug target for medicinal compounds. GTPase HRas protein may be the target protein of most active compounds which are more thermodynamically stable than GTP within binding site which may prevent entry of GTP and signal transduction for cell formation may be stopped. Most active compounds may be safer to be used after further experimental validation. The study proposed that GTPase HRas protein may be the possible target protein of bis-pyrimidine compounds with better selectivity index.
